# A Review of Factors Affecting Radiation Dose and Image Quality in Coronary CTA Performed with Wide-Detector CT

**DOI:** 10.3390/tomography10110127

**Published:** 2024-10-30

**Authors:** Yihan Fan, Tian Qin, Qingting Sun, Mengting Wang, Baohui Liang

**Affiliations:** 1 School of Medical Imaging, Bengbu Medical University, Bengbu 233000, China; fyh20241017@163.com (Y.F.); 20221010019@stu.bbmc.edu.cn (T.Q.); sun156764@163.com (Q.S.); 2 The Second Hospital of Anhui Medical University, Hefei 230000, China; wmtlyh@126.com

**Keywords:** wide-detector CT, coronary computed tomography angiography, radiation dose, image quality

## Abstract

Compared with traditional invasive coronary angiography (ICA), coronary CT angiography (CCTA) has the advantages of being rapid, economical, and minimally invasive. The wide-detector CT, with its superior temporal resolution and robust three-dimensional reconstruction technology, thus enables CCTA in patients with high heart rates and arrhythmias, leading to a high potential for clinical application. This paper systematically summarizes wide-detector CT hardware configurations of various vendors routinely used for CCTA examinations and reviews the effects of patient heart rate and heart rate variability, scanning modality, reconstruction algorithms, tube voltage, and scanning field of view on image quality and radiation dose. In addition, novel technologies in the field of CT applied to CCTA examinations are also presented. Since this examination has a diagnostic accuracy that is highly consistent with ICA, it can be further used as a routine examination tool for coronary artery disease in clinical practice.

## 1. Introduction

Coronary artery disease (CAD) is one of the globally prevalent cardiovascular diseases, which continues to be the leading cause of death in both developed and developing countries [1]. Currently, various medical imaging techniques, such as invasive coronary angiography (ICA), coronary computed tomography angiography (CCTA), and intravascular ultrasound (IVUS), are utilized to diagnose CAD [2]. Traditionally, ICA is regarded as the gold standard for diagnosing CAD [3,4] but its invasiveness and high expenses limited its broad application. In recent years, the rapid development of CT technology has resulted in multi-row detector CT with increasing spatial and temporal resolution and pushed CT imaging toward ultra-precision and functionalization [5,6]. In this case, small structures such as branches of the coronary arteries can be clearly differentiated. Plentiful evidence has shown the advantages of CT imaging in diagnosing CAD. Consequently, CCTA is becoming more widespread in clinical practice and has served as a robust and reliable imaging tool for physicians to evaluate CAD.

In 1999, Becker et al. successfully used 4-detector row CT systems for the first time for coronary artery examination; however, due to the slow scanning speed of the machine, the injection time of the contrast agent was required to be longer, which led to the need for the patient to hold their breath for a long time [7]. It should also be noted that the spatial resolution obtained was relatively low, making it challenging to generate CT images that qualify for diagnosis. Following the introduction of the 64-row CT scanner in 2004, which significantly improved the diagnostic accuracy of CAD, the evaluation of coronary artery stenosis shifted from the use of ICA to CCTA [8]. One of the newest types to emerge in 2005, dual-source CT, is equipped with two sets of X-ray tubes and detectors arranged orthogonally, allowing for a further increase in the temporal resolution. In 2007, there was a breakthrough in Z-axis beam width. The Aquilion One scanner and Revolution CT scanner are both equipped with 160 mm wide detectors in the axial direction. Both of them enable whole heart imaging in a single cardiac cycle without table motion, thus reducing the scanning duration and eliminating the presence of stair-step artifacts common in 64-row CT [9,10,11,12]. Numerous studies have confirmed that overexposure is associated with greater induction of malignant tumors [13,14,15]. According to the “As Low As Reasonably Achievable” principle, the optimization of CCTA protocol is vital to alleviate concerns about patients’ radiation burden. Therefore, this article initially reviews the technical parameters of wide-detector CT that allow performing CCTA in patients with complicated conditions and then describes the improvements they have made to enhance image quality and minimize radiation exposure. Finally, new advances in CT technology applied to CCTA examinations are presented.

## 2. The Hardware Configuration of Wide-Detector CT

In a coronary CT examination, the length of the cardiac anatomy to be covered typically ranges from 120–140 mm. With conventional 64-row CT scanners featuring Z-axis collimation around 20–40 mm, the X-ray beam necessitates 5–8 cardiac cycles to encompass the entire heart, leading to stair-step artifacts at the image junctions. To cope with this challenge, various manufacturers have researched wide-detector CT. In general, detector rows in the Z-axis larger than 64 rows are considered wide-detector scanners, including 128-, 256-, and 320-row CT scanners, thus covering the entire heart in one or two cardiac cycles to be less dependent on patients’ heart rate during CT scanning [16]. This new wide-detector scanner has great potential for the clinical routines. The wide-detector CT hardware configurations of various vendors routinely applied to CCTA scans are shown in Table 1.

## 3. Comparison Between Wide-Detector CT and Dual-Source CT (DSCT)

The coronary arteries move rapidly and complexly throughout the cardiac cycle; thus, an excellent temporal resolution of the CT scanner is essential for obtaining artifact-free images. Several manufacturers have chosen to increase the number of detector rows in the Z-axis to address such challenges and reduce scanning time. However, in this case, the X-ray beam will have a more significant divergence angle, thus leading to cone-beam artifacts, which will require more complex reconstruction algorithms to resolve later [23]. Another promising alternative is the DSCT proposed by Siemens, which, by means of two X-ray tubes and detectors arranged at an angle of approximately 90°, allows temporal resolution to be reduced to a quarter of the gantry rotation time. In addition, compared with other scanners, the second-generation DSCT is equipped with a prospectively ECG-triggered high-pitch scanning with a selectable maximum value of 3.4, which realizes cardiac acquisition within 250 ms [24]. Although this imaging modality currently features the lowest radiation dose and the least amount of contrast used, it is more susceptible to heart rate and motion artifacts, making it more suitable for patients in sinus rhythm with low heart rates [25].

In conclusion, wide-detector CT and DSCT are two solutions proposed by different manufacturers to improve temporal resolution. With the rapid development of hardware and software technology, both enable obtaining images that meet diagnostic requirements at any heart rate. From a radiation protection point of view, a prospectively ECG-triggered high-pitch scanning is preferred for patients in sinus rhythm with low heart rates [26,27,28].

## 4. Factors Affecting CCTA Image Quality and Radiation Dose

**Figure 1:**
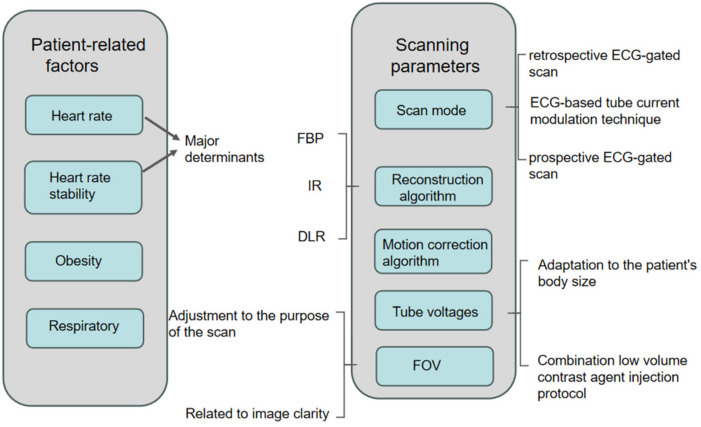
Diagram showing factors affecting image quality versus radiation dose for CCTA.

In this article, the factors affecting the radiation dose and image quality of CCTA examinations will be divided into two parts, as shown in Figure 1:

### 4.1. High Heart Rate and Arrhythmia

**Figure 2:**
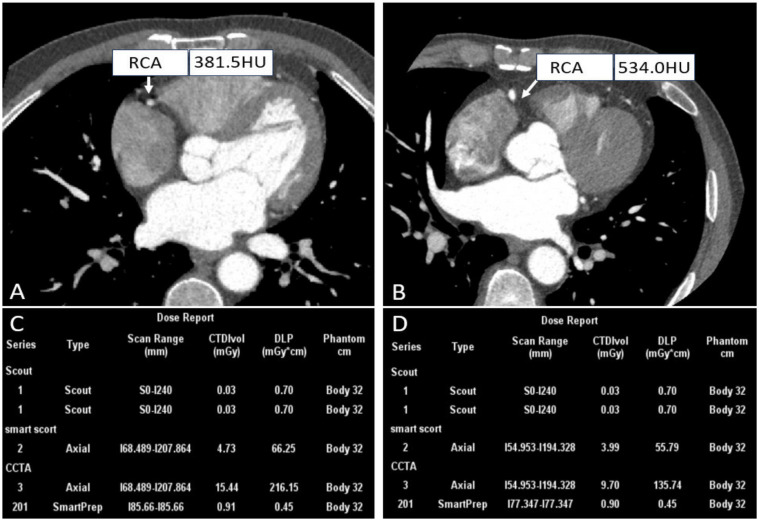
Right coronary CTA images and dose reports of patients with different heart rates. ((**A**,**C**) A patient with a heart rate of 228 bpm; (**B**,**D**) A patient with a heart rate of 71 bpm).

Whether CCTA is successful depends on various factors, among which heart rate is a major determinant. Specifically, the motion artifacts caused by rapid cardiac motion and arrhythmias impact clinical diagnosis. In early 4-row and 16-row CT, a heart rate of less than 75 beats/min (bpm) was a prerequisite for obtaining excellent images [29,30,31]. Patients with high heart rates usually need to administer β-blockers to lower their heart rates before undergoing CCTA for high-quality images. Despite its advantages, 5–11% of patients still have contraindications to this premedication, and 25–30% of patients experience insufficient heart rate reduction even though they take betablockade [32]. To overcome this obstacle, given that the wide-detector scanner with respect to the superior temporal resolution coupled with motion correction algorithms, thus the ability to adequately “freeze” cardiac motion offers an opportunity to image patients that previously posed challenges to CCTA, further producing motion-free images, as shown in Figure 2. 

This is based on a prospective study of 90 patients with a uCT960+ scanner developed by United, which aims to investigate the feasibility of axial scanning in patients with high heart rates. In this case, ePhase and CadioCapture were used to select the best phase and suppress artifacts [22]. It was found that when both techniques were used together, the diagnostic segment number increased to 99.8% with minimum radiologist involvement. 

Similarly, Wang et al. exploited the wide-area coverage of an Aquilion One CT scanner and performed prospective ECG-gated scans in patients with different heart rates [33]. It needs to be noted that heart rates were negatively correlated with image quality while positively correlated with radiation dose. The specific reasons are due to the fact that in patients with heart rates < 70 bpm, only one cardiac cycle of data needs to be acquired, whereas in patients with high heart rates, the temporal resolution of a single cardiac cycle is not sufficient to freeze cardiac motion, and the acquisition of two or three cardiac cycles is required in conjunction with the multisegmented reconstruction to achieve improvement of resulting image. 

Snapshot freeze (SSF) is a GE-developed motion correct algorithm that utilizes adjacent cardiac phases within a single cardiac cycle to characterize the motion velocity and path, positioning the coronary arteries precisely at the target phase while reducing reconstruction time [34]. In terms of clinical routine and scientific research, most studies have investigated improving the novel algorithm on image quality, among which Chen et al. focused on patients with high heart rate variability (HRv). In their retrospective study, according to cardiac rhythms during scanning, 166 patients with uncontrolled heart rates were divided into two groups (group A: HRv ≤ 10 bpm, group B: HRv > 10 bpm), and CCTA was performed with auto-ECG gating on Revolution CT. In addition, SmartPhase and SSF techniques were applied to select the optimal reconstruction phase and motion correction with the aim of evaluating the feasibility of performing single-heartbeat CCTA. Although there were no statistically significant differences between the two groups with regard to objective and subjective image quality, the radiation dose in patients with more severe arrhythmias was higher [35]. The higher radiation dose was due to the scanner automatically widening the acquisition window in patients with severe HRv to provide more options for image reconstruction.

In conclusion, while tachycardia and arrhythmia have been the key factors affecting the success rate of CCTA scans, the advent of the wide-detector CT has allowed them to be performed without medication aid.

### 4.2. Scanning Mode

Since the coronary arteries move quite rapidly and complexly throughout the cardiac cycle, it is relatively challenging to capture heart images that are suited for clinical diagnosis. In this regard, cardiac gating technology needed to be applied during scanning, which allows the resulting images to be localized to specific parts of the cardiac cycle, potentially mitigating cardiac motion artifacts. There are two particular types of ECG-gated techniques: retrospective ECG-gating and prospective ECG-gating scanning. The former choice is regarded as a more traditional scanning modality, in which a spiral acquisition with a pitch of less than one is used, with the X-ray tube being switched over the whole heart phase, allowing continuous spiral scans and synchronized information on the cardiac motion.

Given the above scanning characteristics, radiologists can select the cardiac phases with better image quality for image reconstruction on the workstation after scanning [36]. In addition, the information obtained for the entire cardiac cycle allows the analysis of cardiac function but also results in a higher radiation dose to the subject. 

Compared with retrospective ECG-gated CCTA, the ECG-based tube current modulation technique is an effective modality of reducing the radiation dose based on the reality that coronary arteries move slowly during late systole and mid-to-late diastole. Therefore, the higher tube current is generally maintained during diastole, with lower tube currents selected for the rest of the period [37]. This method enables the reduction of radiation dose without compromising image quality. Still, it is not applicable to patients with severe arrhythmia, as the exposure phase with the maximum tube current in the cardiac cycle is preset before scanning; changes in the heart rate during the tube exposure may lead to a decrease in the tube current at the desired reconstruction phase, which may affect image quality for diagnosis [38]. 

To investigate the effectiveness of this technique in dose management, Hausleiter et al. compared cardiac CT scans with and without an ECG-based tube current modulation technique at 120 kV with 64-slice CT systems and found that the manner achieved dose reduction of 37% with no difference in image noise [39]. Another initiative to lower the exposure dose of cardiac CT is the application of a prospective ECG-gating scan, where not only step-and-shoot mode is employed to avoid helical oversampling but also X-ray exposure is performed at predefined time-points of the cardiac cycle, generally in late diastole [40]. In the process of axial scanning, after the tube is rotated around the subject, the table is moved to the next position, and data acquisition continues in the same phase selected for the R-R interval until image data are acquired for the entire coronary artery region of the heart. It is worth noting that no X-rays are generated during the movement of the table, so the radiation dose delivered by the patient is significantly reduced. The disadvantage of the axial format is an inability to assess cardiac function due to the lack of image acquisition in the remaining cardiac phase. In addition, the image quality resulting is more vulnerable to heart rate and motion artifacts, thus limiting this scanning modality to patients in sinus rhythm with heart rates of ≤65 bpm [41]. 

In a recent study, Tang et al. evaluated image quality and radiation exposure of CCTA in a cohort of 400 children. These subjects were divided into a control group and a study group to investigate the feasibility of prospective ECG-gated multiphase scans on children with various heart rates where children in the control group underwent retrospective ECG-gated scanning technology [42]. Each was further divided into four subgroups based on heart rate frequency to ensure the persuasiveness of the experiment. It is noted that there was no significant difference in coronary artery image quality between the two groups at the same heart rates, and a dramatic reduction in radiation dose (72%) has been achieved, with excellent popularization significance. 

In conclusion, prospective cardiac gated axial scanning enables a substantial reduction in radiation dose without sacrificing image quality compared with retrospective cardiac gating, but the method restricts the patient to sinus rhythm and a low heart rate (i.e., less than 65 or 70 bpm). ECG-based tube current modulation techniques are equally unsuitable for patients with irregular heart rates and offer the same advantages in terms of radiation dose reduction compared to standard retrospective ECG-gated protocols.

### 4.3. Reconstruction Algorithm

Over the past years, CT image reconstruction has mainly relied on the filtered back projection (FBP) algorithm; however, the process usually involves high-pass filters, which will accentuate the noise and streak artifacts, thus leading to less satisfactory image quality at low currents [43,44]. In contrast, iterative reconstruction (IR) is recognized as an alternative method to FBP, allowing noise reduction while maintaining comparable radiation doses or delivered dose reduction while maintaining equivalent image quality. One of the concerns with the IR algorithm, which involves multiple iterative cycles, is the amount of time-consuming generating final output images. Due to increased computational power, each CT manufacturer has developed proprietary IR methods used for CCTA, trying to achieve a trade-off between image quality and radiation dose in clinical CT images, as summarized in Table 2. At some level, using IR algorithms can compensate for the adverse effects of dose reduction strategies. To the best of our knowledge, numerous studies have been conducted on the IR used for CCTA scans in recent years. The specific details are described below:

Toshiba has developed the adaptive dose reduction (AIDR), the three-dimensional AIDR (AIDR 3D), and the forward-projected model-based iterative reconstruction solution (FIRST), of which AIDR 3D is currently routinely configured in all major multi-detector CT scanners from this manufacturer [45]. In a study investigating the impact of AIDR3D and FBP on image quality, 100 subjects were randomly enrolled in coronary evaluation by Fareed et al. using a 320-detector row CT scanner (Aquilion ONE, Toshiba Medical Systems). The results were assessed in the same individuals to eliminate the effects of interindividual factors, showing lower noise levels and improved SNR and CNR with the application of AIDR3D, as well as a higher subjective image quality score [46]. 

The iDose4 and iterative model reconstruction (IMR) introduced by Philips are also favorable regarding radiation dose reduction. In one study, 98 patients with an indication for CAD were randomly assigned to the study group (80 kV; automated-mAs; 60 mL of CM, 350 mg/mL; IMR) and the control group (100 kV; automated-mAs; 70 mL of CM, 400 mg/mL; iDose4). The results subsequently demonstrated that low-dose CCTA studies combined with IMR reconstruction allowed for high-quality images and a significant reduction in radiation dose exposure (38%), in contrast to standard CCTA protocols with iDose4, which could be used to perform CT dose optimization protocols [47]. 

In order to solve the problem of pronounced image noise of FBP with low tube current, GE developed adaptive statistical iterative reconstruction (ASIR) and model-based iterative reconstruction (MBIR) algorithms. It is worth mentioning that ASIR was the first IR applied to cardiac CT and is also considered a hybrid IR algorithm because of its ability to be mixed with FBP [48]. More recently, the effect of contributions of ASIR on image quality was studied in a 3D-printed cardiac insert and Catphan 500 phantoms. Specifically, image reconstructions were performed using FBP and 40% and 60% ASIR at 100 kV and 120 kV, respectively. The best low-dose cardiac CTA results were obtained when a level of 60% ASIR was combined with 100 kV, comparable to the standard CCTA scanning protocol (120 kV + 40% ASIR), but with the possibility of a 50% dose reduction. Consequently, they concluded that combining a higher IR reconstruction algorithm strength with a lower tube voltage enables an effective reduction of the radiation dose, and it could be considered for further application in clinical practice [49]. In addition, Benz et al. conducted several clinical studies validating the effectiveness of MBIR in dose optimization. Among 91 subjects consecutively undergoing ECG-gating axial scans, compared to the incremental blending of ASIR (20%, 40%, 60%, 80%, and 100%), MBIR performed the best noise reduction and higher SNR in the left main artery and right coronary artery, which is expected to control the radiation dose in sub-millisieverts [50]. ASIR-V is a novel algorithm between ASIR and MBIR, further introduced by GE in 2014, comparable to ASIR regarding reconstruction speed and MBIR regarding image quality [51]. In a CCTA study of 65 subjects, Benz et al. compared the effect of different levels of ASIR-V (20%, 40%, 60%, 80%, and 100% ASIR-V) with the FBP reconstruction on the image quality performed on the Revolution scanner, and ultimately found that 100% ASIR-V had the best noise reduction and image quality, allowing for its use in low-dose CCTA scanning protocols (0.49 mSv) [52]. 

In recent years, deep-learning reconstruction (DLR) algorithms have been developed to reduce image noise by utilizing deep-learning systems to train convolutional neural networks at low radiation doses in order to reconstruct CT images at regular doses [53,54]. DLR algorithms currently used in clinical practice involve AiCE (Canon Medical), TrueFidelity (GE Medical), and Precise Image (Philips Medical) [55]. Multiple studies have consistently demonstrated the ability of DLR algorithms to reduce image noise and, therefore, radiation dose. A comprehensive study of the effect of five available reconstruction methods (FBP, AIDR-3D, FIRST Cardiac, FIRST Cardiac sharp, and DLR) on CCTA showed a significant improvement in quantitative image quality with the aid of DLR while maintaining comparable diagnostic performance [56]. In terms of dose optimization, improved image quality with reduced delivered dose has been validated with DLR compared with ASiR-V 70% and ASiR-V 100% in a total of 50 patients undergoing two axil CCTA scans. Studies with lower-dose scans and DLR reconstruction have shown better image quality than normal-dose imaging with ASiR-V 70% and ASiR-V 100%. It is worth mentioning that an improved scan protocol has shown a 43% reduction in radiation dose, and the image noise was at least comparable to a standard scan with ASiR-V 100%, both being significantly lower than ASiR-V 70%, without significant differences in stenosis severity, assessment of plaque composition, and plaque volume quantification [57]. 

In conclusion, IR algorithms developed by various manufacturers can further reduce the radiation dose of CCTA examinations while ensuring image quality, and DLR algorithms developed based on artificial intelligence further improve image quality and dose optimization.

### 4.4. Tube Voltages

**Figure 3:**
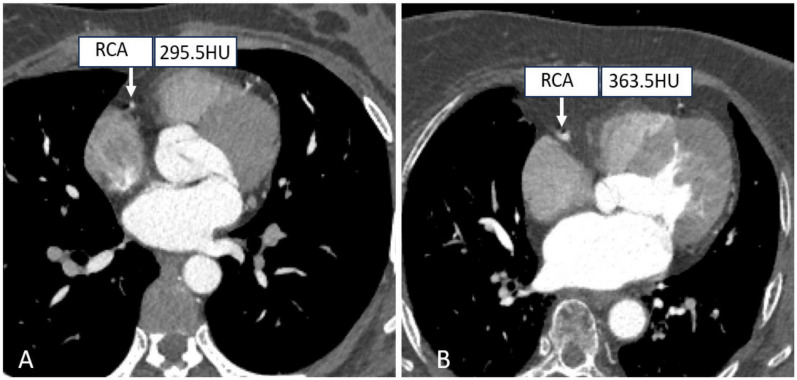
Right coronary CTA images with different tube voltages. ((**A**) A patient with a tube voltage of 100 kV; (**B** A patient with a tube voltage of 120 kV.).

Tube voltages have been found to play an important role in dose-saving strategies. On the one hand, in the case of a particular tube current, the radiation exposure is proportional to the square of the tube voltage. On the other hand, coronary vascular enhancement will be better due to higher attenuation levels of iodinated contrast medium acquired at low kV-setting, which increases the contrast between the artery lumen and the surrounding tissues, potentially decreasing the volume of contrast agent used [58,59]. Thus, an effective strategy to minimize tube voltages reasonably while maintaining the image quality is highly desirable, as shown in Figure 3. In addition, it was previously reported that the incidence of acute kidney injury after intravenous injection of contrast media ranges from 2% to 35%, making it necessary to minimize the tube voltage as much as possible without compromising the quality of the diagnostic images during the clinical routine CT examinations [60]. 

Traditionally, a tube voltage setting of 120 kV is selected as the standard reference for CT scanning protocols, but in some cases, 100 kV or 80 kV is recommended for cardiac CT examinations in non-obese patients. Such a suggestion is supported by several studies. An earlier work by Tan et al. aimed to study the effect of tube voltage on image quality and radiation dose with prospective ECG-gated CCTA at 80, 100, and 120 kV; thus, a comprehensive systematic search of PubMed, the Cochrane Library, CINAHL, Web of Science, ScienceDirect, and Scopus was performed without publication limitation. It was found that radiation dose reductions of 38% to 83% at 80 kV and 3% to 80% at 100 kV could be achieved with preserved image quality, explaining the feasibility of a low tube voltage setting in prospective scanning [61]. In another study, using prospective ECG-gating CCTA on patients with a body mass index (BMI) ≤ 30 kg/m^2^, Wang et al. reported a further reduction in effective radiation dose of 7.2 ± 5.8 mSv versus 3.1 ± 2.1 mSv in standard (120 kV, 320 mg I/mL) versus optimized scan protocol (100 kV, 270 mg I/mL) with no impact on diagnostic purposes [62]. 

In conclusion, in CT examinations, the tube voltage should be adjusted taking into account the patient’s body size. For patients with normal BMI, a scanning setting with low tube voltage and low concentration of low-dose contrast with IR algorithms [63] can effectively reduce the radiation dose and iodine burden while ensuring image quality and minimizing the risk of kidney impairment to some extent.

### 4.5. Scanning Field of View (SFOV)

**Figure 4:**
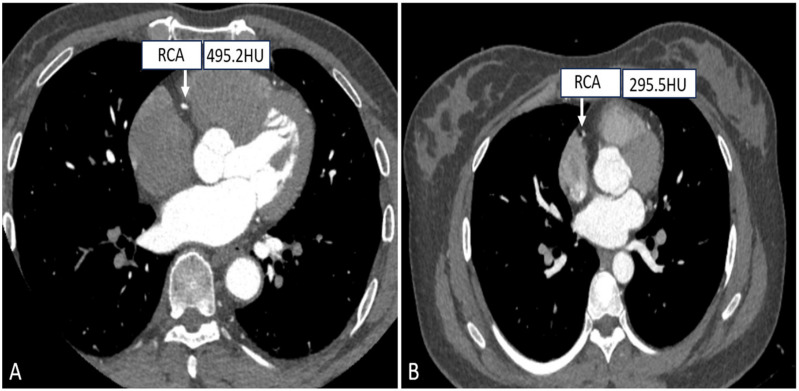
Right coronary CTA images with different SFOV. ((**A**) A patient with small SFOV; (**B**) A patient with large SFOV.).

As it is known to us, the scanning field is positively correlated with the CT radiation dose, and accurate settings of the FOV can effectively control the radiation dose. Small FOV scanning protocols mainly consider the heart as the organ of interest, while the lungs, pleura, and mediastinum are not entirely included, and for patients who need to examine the heart and the entire chest, a choice of large FOV (300–500 mm) is available for simultaneous evaluation of the coronary arteries, pulmonary arteries, and thoracic aorta in chest pain protocols, as depicted in Figure 4 [64]. Muenzel et al. investigated the effect of the small FOV group (FOV ≤ 250 mm) versus the large FOV group (250 mm < FOV ≤ 400 mm) on image quality and radiation dose, and the results showed that the average effective dose of the small FOV was reduced from 4.8 mSv to 3.9 mSv as compared to the large FOV, while there was no significant difference in image quality [65]. 

In conclusion, in order to further optimize the radiation dose, a small FOV can be selected in relation to the patient’s body size and the purpose of the examination, which also improves image clarity.

## 5. Diagnostic Value

ICA is the most accurate method for diagnosing CAD; however, conventional coronary angiography requires mechanical insertion into the coronary arteries, which can lead to cardiovascular complications and localized arterial puncture site complications, increasing the risk of invasive examination [66,67]. In contrast, CCTA is a minimally invasive examination that plays an important role in exclusive CAD and is more sensitive than ICA for detecting atherosclerotic plaques [68]. Although CCTA does not recognize plaque rupture or erosion features, it can detect plaques larger than 1 mm and classify them as calcified, noncalcified, or mixed [69,70]. 

Hou et al. used 256-row CT to confirm the high reliability of CCTA in the diagnosis of suspected CAD, with diagnostic accuracy highly consistent with ICA [71]. Similarly, de Graaf et al. performed CCTA and invasive angiography in 64 patients using the Aquilion One scanner, a 320-row CT, and when four images that could not be used for diagnostic purposes were excluded, the negative predictive value of the CCTA technique was 100%. The diagnostic accuracy was 95% at the patient level for the detection of ≥50% stenosis. For the assessment of ≥70% stenosis, the negative predictive value was 98%, and the diagnostic accuracy was 95%, which suggests that the CCTA with 320-row CT allows for an accurate, noninvasive evaluation of CAD [72]. Given the economic, rapid, and easy-to-use characteristics of the multislice CTA technique, CCTA can be used as a means of routinely ruling out CAD, variations, and malformations in clinical examinations.

## 6. New Technologies in the Field of CT

All of the above are the effects of parameter settings on overall image quality, radiation dose, and diagnostic accuracy. With the rapid development of multi-row detector technology, CCTA is not limited to the anatomical level; functional CCTA can assess ventricular function, valve motion, perfusion, etc. [73]. In particular, noninvasive fractional flow reserve from coronary CT angiography (FFR-CT) is a novel coronary heart disease testing technique that is not only used to assess the physiologic significance of coronary artery stenosis but also serves as a decision criterion for coronary revascularization in clinical practice [74]. Specifically, the technique is performed based on CCTA images for post-processing to create a three-dimensional model, and then computational fluid dynamics (CFD) modeling is applied to simulate coronary blood flow. By combining it with other traditional imaging metrics in clinical practice, there is potential for better prediction of major adverse cardiovascular events (MACE). Recently, a study [75] evaluated the accuracy of FFR-CT at different levels of image quality and found that it was only related to motion artifacts and nitroglycerin dose taken before the scan and was not significantly affected by patient- or technical-related factors. 

In addition, in the past decade, photon-counting computed tomography (PCCT), introduced by Siemens, has become the most influential technology in the field of CT imaging. Based on a new generation of X-ray detectors, the PCCT allows for multi-energy imaging, lower noise image reconstruction at low doses, improved spatial resolution and soft-tissue contrast, further decreases in radiation exposure, and optimizes the use of contrast agents [76]. In a prospective study, 68 patients with severe aortic valve stenosis and referral for pre-transcatheter aortic valve replacement (TAVR) were enrolled. Taking ICA as a reference standard, all participants were examined using a dual-source PCCT scanner, and it was found that PCCT demonstrated high sensitivity, specificity, and accuracy in terms of the ability to determine coronary artery stenosis ≥50% on a participant-, vessel-, and segment-based level [77]. It is believed that photon-counting CCTA provides higher diagnostic accuracy of CAD in high-risk populations with the prospective to dramatically change the use of CT in cardiovascular imaging in the coming years while reducing patient referrals to ICA.

## 7. Conclusions and Outlook

The rapid development of CT technology leads to a greatly improved success rate of coronary imaging and has practical application value in the early exclusion of CAD. Meanwhile, many facts and studies have been published that depict increased induction of malignancies associated with exposure. Under this circumstance, this paper proposes appropriate scanning techniques.

First, high heart rates and rhythm variability have always been a challenge for CCTA, and wide-detector CT has made it possible to perform the scan in these patients even when they were not taking medications to control their heart rate. However, in patients with high heart rates and arrhythmia, the machine will automatically recognize the adjacent phases of the scan for image reconstruction, resulting in a higher radiation dose. Clinical control of the patient’s heart rate before the examination should be performed as much as possible to improve the examination’s success rate and optimize the radiation dose. Subsequently, the choice of scanning mode, reconstruction algorithm, tube voltage, and SFOV is closely related to radiation dose and image quality. Familiarity with the technical parameters of wide-detector CT from various vendors and the factors affecting radiation dose in CCTA examinations can help personalize the scan to the patient and optimize the dose to the greatest extent possible.

## Figures and Tables

**Table 1:** Hardware configurations of various manufacturers are routinely applied to CCTA scans.

Vendor	Canon			GE	Philips	United		Neusoft	
CT System	Aquilion ONE [17]	Aquilion ONE Vision [18]	Aquilion ONE Genesis [19]	Revolution CT [20]	Brilliance iCT [21]	uCT960+ [22]	uCT968	NeuViz Epoch+ CT	NeuViz Glory+ CT
Number of Detectors Rows	320	320	320	256	128	320	320	256	128
Detector Z-axis Coverage	160 mm	160 mm	160 mm	160 mm	80 mm	160 mm	160 mm	160 mm	80 mm
Detector Element	0.5 mm	0.5 mm	0.5 mm	0.625 mm	0.625 mm	0.5 mm	0.5 mm	/	0.625 mm
Rotation Time	350 ms	275 ms	275 ms	280 ms	270 ms	250 ms	/	235 ms	235 ms
Temporal Resolution	175 ms	137 ms	137 ms	140 ms	135 ms	/	/	/	/
Iterative Reconstruction	AIDR3D	AIDR3D	AIDR3D, DLR	ASiR-V	iDose4	/	AIIR	ClearInfinity	
Modulation Technique	Expusure SURE			Smart mA kV assist	DoseRight	/		Intelligent mA technology Auto-kV	

**Table 2:** The present reconstruction algorithms applied to coronary imaging.

Vendor	Canon	GE	Philips
IR Algorithm	Adaptive dose reduction (AIDR)	Adaptive statistical iterative reconstruction (ASIR)	iDose4
	Three-dimensional AIDR (AIDR3D)	Model-based iterative reconstruction (MBIR)	Iterative Model Reconstruction (IMR)
	Forward-projected model-based iterative reconstruction solution (FIRST)	Adaptive statistical iterative reconstruction-V (ASIR-V)	
DLR Algorithm	AiCE	TrueFidelity	Precise Image
